# On the Treatment of White Swellings, &c.

**Published:** 1859-01

**Authors:** 


*On Immediate Straightening and Cauterization under Starch-bandages, in the Treatment of White Swellings. Utility of Chloroform in Discriminating between Muscular Contraction and Coxalgia. Croup; Cutaneous Anæsthesia. Tubing of the Glottis, substituted for Tracheotomy.*

A communication made to the Academy of Sciences by Dr. Bonnet, of Lyons, on the treatment of white swellings by instantaneous straightening, has, in latter times, created a very lively sensation in the medical world.

In many diseases of the joints, says Dr. Bonnet, articular lesions coëxist with deviations and incomplete luxations. Prudence sometimes points out he propriety of not interfering with these mal-formations, but it is often tequisite to replace the limb in its proper direction. Now, when straighten
r ing is necessary, there are two modes of effecting it; immediate straightening by forcible extension, and slow and gradual straightening by machinery. Great experience in both, which he has compared, has proved to Mr. Bonnet the superiority of the former of these modes over the latter.

Dr. Bonnet already proclaimed, seven years since, its excellency in coxalgia attended with fibrous adhesions. This surgeon now shows that in all deformities without organic lesion, or coëxistent with rheumatic, or scrofulous white swelling in progress of increase, or resolution, the mode of straightening to be preferred, is that which requires but one operation, followed by the protracted immobilization for several weeks.

The essential and general rule to be followed in such cases is, first to loosen the articulation during artificial anæsthesia, and to restore its mobility completely. This may be accomplished by an alternate series of gentle flexions and extensions, graduated and carried to the extreme limit of the natural movements. The adhesions being destroyed and mobility restored, the straightening of the deformity and the reduction of the displacement may be proceeded with. Proper tractions and pressure are then sufficient, and success is in proportion to the mobility obtained.

When the limb operated on has resumed the best possible direction, nothing further is required but to fix it in its new position with all due precaution, in order to prevent or alleviate the consequent pains. Grooves constructed with annealed iron-wire, properly lined, may be employed for this purpose. But these grooves are not indispensable, and, in Dr. Bonnet's estimation, it is preferable to use a wadded and starched pasteboard bandage. Some days ago, Mr. Bonnet applied his apparatus, in the presence of a great number of persons, at the clinical lecture of Dr. Nélaton; and we remarked the minute care with which he arranged its various parts. The surgeon first rolled round the limb thick strips of wadding, which he fixed in their places by a few turns of a linen roller; pasteboard splints, impregnated with liquid starch paste were placed over it, and were, in their turn, covered witb starch-bandages of considerable length; in order to give this apparatus immediate solidity, Mr. Bonnet applied over all, annealed iron-wire splints, which he prefers to Mr. Seutin's dry pasteboard ones.

Thus constituted, the starch-bandage must be left in its place for three weeks or a month. At the end of that time it is removed, the diseased parts are examined, and the surgeon, by applying either a new bandage of the same nature or some other apparatus, completes the straightening, and endeavors to obviate the return of the deformity, which long preserves a great tendency to recur.

But how brilliant soever the result of the straightening may be, when viewed with reference to form, to functional aptitude, or to the rapid improvement of the inflammatory state and the removal of pain, it does not, however, directly tend to cure the disease itself. To obtain this ultimate benefit, Mr. Bonnet practices cauterization under the starch-bandage.

This cauterization can be performed with caustic potash, Vienna paste, or chloride of zinc. Mr. Bonnet usually employs potash lozenges wrapped up in wadding, so that the escharotic liquid may not extend beyond the point to be acted on.

Whatever caustic may be selected, it is important that the bandage applied after cauterization should extend far enough to procure absolute immobility and a complete protecting cover. Thus, for instance, after an operation on the knee, the bandage should extend from the extremity of the foot to the pelvis, and thus render motionless the foot and even the hip. In this manner the counter irritants act exclusively on the skin and the cellular tissue, without the local inflammation which follows the application of caustic being communicated to the diseased synovial membranes, as would happen were the limbs abandoned to their natural movements.

Dr. Bonnet began, in the spring of 1857, cauterizations in combination with immobility and occlusion, and since that period, sixty cases, referring to white swellings of the foot, knee, elbow and hip, have testified in favor of this method. In the fifteen months which have just expired, Dr. Bonnet has cured, or improved to a degree bordering on cure, three white swellings of the foot, as many of the knee, and one of the elbow, all attended with numerous abscesses proceeding from the joints and in conditions which, according to habitual surgical practice, would have justified amputation.

We should add that, during the period of cicatrization of the cauterized parts, the limbs remain supported in grooves which, while they insure immobility, expose to view the regions which require to be dressed. At the same time, a treatment calculated to modify the general state of the patient is instituted, and during the convalescence, light supports are used, which can be placed and removed at pleasure, an indispensable prop to limbs weakened by too long protracted inaction. Such is the method expounded by Dr. Bonnet, not only before the Academy of Sciences, but before the greater part of the learned societies of Paris. Several members of the Society of Surgery expressed a desire that Mr. Bonnet should state with precision the circumstances in which immediate straightening may be practiced in coxalgia. Mr. Bonnet replied that for four months past he had attempted straightening eight times in that articular disease, and that he had succeeded seven times. He attributed this enormous proportion of success to the fact of having operated on subjects under fifteen years of age. Before the twelfth year, straightening, applied to coxalgia, presents chances of success so numerous as almost to amount to certainty, unless the deformation is of several years' standing and presents many closed sinuses. Above the age of fifteen, the difficulties of straightening are extreme, particularly if the injury is more than six months' date. The effects of counter-irritant cauterizations are then but uncertain, and deep and direct cauterizations may be attended with danger. Relatively to the circumstance of the disease being acute or chronic, Mr. Bonnet has always found that, far from being counter-idicated by the acute state, straightening and immobilization are the best means of treatment which can be opposed to the inflammatory action. In the chronic period, straightening in children is still applicable, when any traces of mobility remain. Complete anchylosis, at any age, and in every case, is a formal counter-indication to straightening. To confute the objections raised on the subject of the inflammatory accidents, which might be induced in a diseased articulation by his operation, the skilful surgeon of Lyons had but to invoke his own experience. By resorting to methodical movements alone, by keeping up a uniform temperature around the diseased limb by means of the thick layer of wadding with which his apparatus is provided, by rendering the limb immovable after it has been straightened, Dr. Bonnet has never had to deplore any serious accident, even when, to attain his object, he has been compelled to perform the subcutaneous section of the contracted muscles.

—We shall certainly revert to a question which promises to afford for a length of time matter for discussion at the meetings of our learned societies; but we have deemed it a duty at once to call the attention of our readers to one important result obtained by the application of Dr. Bonnet's method. We allude to the facility with which artificial anæsthesia generally enables the practitioner to discriminate between mere muscular contraction and real coxalgia.

The *Gazette des Hôpitaux* has published on this subject several interesting cases, one of which was observed in Dr. Robert's wards, at the hospital of the Hôtel-Dieu in Paris.

A young woman, twenty-five years of age, occupying the bed No. 3, of Saint-Paul's ward, presented, the last four months, all the symptoms of coxalgia, viz., pain in the hip, improper attitude of the limb, which was bent upon the pelvis, placed in adduction and slightly rotated inwards, with consecutive deviation of the pelvis, immobility, resistance to straightening, attempts to effect which occasioned much pain, etc. Dr. Verneuil, who at present supplies the place of Dr. Robert, desirous of applying Dr. Bonnet's method in this case, had her conveyed to the operating theatre, where, previously to any operation, she inhaled chloroform. Mr. Verneuil expected that he should have to use great strength and he had secured the coöperation of numerous assistants, when, to his surprise, the limb reduced itself, as if spontaneously, at the first efforts of the operator. It was then easy to cause the thigh to perform, without the least violence, the most extensive physiological movements, without experiencing any resistance whatever, and without the hand or the ear detecting the smallest amount of friction. The limb, replaced in its proper position, was maintained by means of Dr. Bonnet's apparatus.

We read on the other hand, in the *Gazette hebdomadaire*, that, in a girl of eighteen, who had been for three years thought to be laboring under coxalgia, anæsthesia, employed for the purpose of immediate straightening, enabled Dr. Robert to ascertain the complete integrity of the coxo-femoral articulation and to discover a muscular contraction, which was most successfully treated by walking, electricity and general tonics.

The same journal relates another fact. well worthy of attention. Dr. Laugier had to treat, in his wards of the Hôtel-Dieu, a boy who had been suffering three years in the right hip. The pain felt by this patient was at times so intense, that for a fortnight he remained seated on the edge of the bed with his feet resting on a chair, his thigh bent and in outward rotation.

Dr. Laugier, unable by ordinary means to relieve this child, put him under the influence of chloroform and performed instantaneous straightening without encountering any serious difficulties; a mechanical apparatus was then applied to render the extension permanent. The pain ceased as it were by magic, and the patient was soon able to walk with crutches.

Facts, such as these, are so much the more deserving of remark, that the muscles, as Dr. Jules Guerin has observed, play an extremely important part in coxalgia. Sometimes they are in a state of contraction, i. e. of spasm, and susceptible of immediate return to their normal length and consistency; at other times they are in a state of retraction or of organic shortening, and do not resume their physiological dimensions unless by laceration or tenotomy. This surgeon even considers muscular contraction the essential symptom, one of the earliest in coxalgia; so that it may exist without disease of the bones, as it, at times, is superadded to a morbid condition of the bones, and is then merely an accessory phenomenon. The benefit which may be derived in these various cases, from an agent that alleviates pain, enlightens diagnosis, and becomes the first element of rational therapeutics, will be readily conceived.

—Within the last six weeks more than twenty children attacked with croup have been operated on at the Saint-Eugenie hospital. The attention of the physicians of this hospital has therefore been much engaged in the observation of this disease; and the clinical studies, to which Dr. Bouchut in particular has devoted himself, have produced results which we deem it our duty to lay before our readers.

We would first notice the existence of a new symptom of croup, which affords an indication for tracheotomy. Since Professor Trousseau has again brought this operation into favor, the question has often been asked at what time, except that of asphyxia with suffocation, the operation should be performed on children attacked with croup. We stated, some years since, in this journal, that Dr. Trousseau was of opinion that it should not take place before the last stage of the disease had fairly set in; more recently the eminent professor has pronounced in favor of an early operation. Increasing asphyxia is, with the major part of practitioners, the determining consideration; but it is known that certain children die with their faces pale, without cyanosis or apnœa; in short without any apparent traces of asphyxia. With regard to the latter therefore, the practitioner has no indication to guide him.

Now there is, in Mr. Bouchut's estimation, a more certain sign of asphyxia, viz. *cutaneous anæsthesia.*

Whether asphyxia be *latent* or *apparent*, when the obstacle to hæmatosis has lasted for some days and the disease is approaching a fatal termination, the skin gradually becomes insensible, and it may be pricked or cut without occasioning any pain, or at least any movement indicative of suffering. If croup requires tracheotomy, it is not rare to see children undergo the operation without manifesting the least sensibility. Dr. Crequy, formerly Dr. Barthez's house-surgeon, has just published in his inaugural thesis the case of a little girl of six years of age operated on for croup, who, having recovered from the operation, declared she had felt no pain. Dr. Demarquay has similarly ascertained the existence of anæsthesia in a woman on whom tracheotomy was performed for an accidental fit of suffocation. Anæsthesia is not therefore an effect of diphtheritis, but of the interruption of hæmatosis, and, as experiments on animals have proved, the result of the presence of too large a proportion of carbonic acid in the blood. Now, what is the clinical importance of this phenomenon? As we have said above, it affords one indication more for the performance of tracheotomy, and this indication will be particularly useful in the case of *latent asphyxia.*

Mr. Bouchut has thus contributed to increase perhaps the favorable chances of this operation. But his ambition did not stop here, and he has recently communicated to the Academy of Medicine an idea which, already carried out with two children attacked with croup, would tend to nothing less than the suppression of tracheotomy as an ultimate resource henceforth useless.

After all the attempts made to arrive at the cure of croup by the introduction of the catheter into the larynx, Mr. Bouchut has drawn from that practice, the principle of a new method, which he designates by the name of *tubing of the glottis*, and which consists in introducing, and leaving for a time in this orifice, a metallic ring.

The instruments he has used twice on living subjects are: 1. curved male catheters of different sizes, open at both ends and intended to penetrate into the larynx as guides to the ring which this organ is to receive; 2. straight cylindrical silver rings, of from $\frac{1}{2}$ to $\frac{3}{4}$ of an inch long, provided at their extremities with two ridges at the distance of a quarter of an inch, and pierced with a hole for the passage of a silk thread, the function of which is to preserve a hold upon the ring from without; 3. a ring to protect the forefinger, or an instrument designed to keep the jaws open. When provided with these instruments, Mr. Bouchut employed them first on a dead subject and he ascertained to his own satisfaction and that of his colleagues that after having been introduced into the larynx, the upper edge of the ring was engaged beneath the superior vocal chord in the ventricles of the larynx; that the movements of the epiglottis and the arytenoid cartilages were not obstructed; that the inferior vocal chord placed itself between the two ridges of the canula, and consequently that it was above the lower ridge corresponding with the internal face of the cricoid cartilage.

This being accomplished, it became necessary to apply the method on the living. An opportunity soon presented itself, but is was during the dreadful epidemic, which in the month of August sent to the Saint-Eugenie Hospital fifteen cases of croup, which terminated fatally. Diphtheritis was generalized; and in addition, as Mr. Bouchut acknowledges, had the two children, on whom the *tubing* was performed, recovered, nothing positive could be concluded from the circumstance. All that can be said, and Mr. Bouchut has kindly permitted us to witness the operation, is that the tubing of the larynx is not a difficult process; that the canula remaining in the glottis for thirty-six hours was perfectly harmless; that the two children could speak distinctly and take liquids without swallowing them the wrong way, and that there was, in every respect, a temporary improvement analogous to that which follows tracheotomy.—*Jour. of Prac. Med. and Surgery.*

